# Post-cholecystectomy Bile Duct Injury in Mexico: Prevention, Diagnosis, Management, and Healthcare System Challenges

**DOI:** 10.7759/cureus.114275

**Published:** 2026-08-10

**Authors:** Tania S Cortes, Leslie-Nahomi Hernandez-Garcia, Luz-Atenas Nieves-Hernández, Andrea Ramírez-Márquez, Daniela De la Vega-Salazar, Marco-Antonio Urbina-Velázquez, Aarón Gonzalez-Espinosa, José-Emiliano González-Flores, Alfonso Sandoval

**Affiliations:** 1 School of Medicine and Health Sciences, Tecnológico de Monterrey Mexico City Campus, Mexico City, MEX; 2 School of Medicine and Health Sciences, Tecnológico de Monterrey México City Campus, Mexico City, MEX; 3 School of Medicine, Universidad La Salle Mexico, Mexico City, MEX; 4 Department of Colon and Rectal Surgery, ABC Medical Center, Mexico City, MEX; 5 Department of General Surgery, Hospital General de México, Mexico City, MEX; 6 Department of Plastic and Reconstructive Surgery, Hospital General de México, Mexico City, MEX; 7 Medical Research and Academic Mentorship, ShareTheMed Research Group, Mexico City, MEX; 8 Faculty of Public Health, Universidad Anáhuac México, Mexico City, MEX

**Keywords:** bile duct injury, biliary reconstruction, cholecystectomy, critical view of safety, hepaticojejunostomy, hepatopancreatobiliary surgery, intraoperative cholangiography, mexico, patient safety, referral pathways

## Abstract

Post-cholecystectomy bile duct injury (BDI) is an uncommon but potentially life-altering complication associated with repeated interventions, impaired quality of life, and substantial long-term morbidity. Although specialized Mexican centers have reported favorable outcomes in complex cases, the national burden, referral patterns, and access to hepatopancreatobiliary expertise remain poorly characterized. This structured narrative review synthesized evidence on the prevention, diagnosis, and management of BDI, with emphasis on the Mexican healthcare system. PubMed/MEDLINE, Embase, Scopus, and Google Scholar were searched for studies published from January 1990 to May 2026. Of 190 records identified, 19 primary studies met the eligibility criteria and were narratively synthesized. BDI appears to result from the interaction of distorted anatomy, visual misperception, unsafe continuation of dissection, technical and platform-related factors, and healthcare-system limitations. Prevention depends on reliable achievement of the Critical View of Safety and timely use of bailout strategies when anatomy cannot be safely defined. Intraoperative imaging may improve anatomical assessment but does not replace surgical judgment. Management should be individualized according to physiological stability, ductal continuity, injury level, contamination, and vascular involvement, with early source control and specialist hepatopancreatobiliary evaluation for major injuries. Mexican evidence is derived mainly from tertiary referral cohorts and suggests delayed recognition, prolonged referral, previous repair attempts, and unequal access to specialized care, despite favorable reconstructive outcomes at selected centers. Improving outcomes will require standardized referral pathways, avoidance of nonspecialist repair, regional centralization of complex reconstruction, and prospective multicenter surveillance.

## Introduction and background

Bile duct injury (BDI) remains one of the most consequential complications of cholecystectomy, given its association with substantial morbidity, mortality, repeated interventions, and persistent deterioration in quality of life. Although its reported incidence is relatively low, ranging from 0.2% to 0.9%, its clinical and epidemiological significance is magnified by the high volume of cholecystectomies performed worldwide [1]. Timely recognition is therefore essential, with intraoperative identification representing the most favorable scenario for accurate characterization of the injury, immediate source control, and appropriate referral or definitive management.

In Mexico, the epidemiological burden of BDI remains incompletely defined because the available evidence is derived predominantly from institutional series conducted at tertiary referral centers, particularly in Mexico City. As a result, current data may not adequately capture national patterns of injury, diagnostic delay, referral, and access to specialized care. In a cohort from the Instituto Nacional de Ciencias Médicas y Nutrición Salvador Zubirán, only 17% of injuries were recognized intraoperatively [2]. Most injuries were diagnosed postoperatively after the development of bile leakage, biliary peritonitis, cholangitis, or abdominal sepsis, frequently following multiple hospital admissions and invasive procedures before definitive reconstruction [2]. Although these findings cannot be extrapolated to the country as a whole, they underscore the need to strengthen intraoperative recognition and highlight the importance of timely access to hepatobiliary expertise, advanced imaging, and interventional resources.

Beyond disparities in infrastructure and resource availability, major challenges include failure to recognize unsafe anatomy, continued dissection despite inadequate anatomical definition, limited access to adjunctive intraoperative imaging, and delayed referral to specialized hepatobiliary centers. Mexican referral series have also reported a substantial proportion of injuries following open cholecystectomy; however, this observation should be interpreted in light of referral patterns, case complexity, and unequal access to minimally invasive surgery. In technically demanding cases, failure to adopt timely bailout strategies may increase the risk of major ductal injury. Moreover, the absence of standardized referral pathways may lead to attempted repair by nonspecialist teams before transfer to an experienced hepatobiliary unit. Such attempts have been consistently associated with greater reconstructive complexity, higher reintervention rates, and poorer long-term outcomes [3,4,5].

The objective of this structured narrative review is to critically synthesize the available evidence on the prevention, diagnosis, and management of post-cholecystectomy bile duct injury, with a particular focus on the Mexican healthcare system. By integrating contemporary international evidence with the limited national literature, this review seeks to characterize the principal epidemiological, clinical, and healthcare system challenges influencing the recognition and management of bile duct injury, while highlighting opportunities to optimize prevention strategies, referral pathways, and multidisciplinary care within the Mexican context.

## Review

Methods

Study Design

This study was conducted as a narrative review supported by a systematic literature search and narrative evidence synthesis. It was not designed as a systematic review or meta-analysis. It focused on BDI following cholecystectomy, with particular emphasis on prevention, diagnosis, therapeutic management, clinical outcomes, and healthcare system challenges relevant to the Mexican context.

For transparency in reporting the literature identification and study-selection process, selected elements of the Preferred Reporting Items for Systematic Reviews and Meta-Analyses (PRISMA) 2020 framework were used where applicable. The review was not intended to claim full compliance with PRISMA 2020. Because substantial heterogeneity was anticipated across study designs, patient populations, injury classifications, interventions, healthcare settings, and reported outcomes, quantitative pooling was not considered appropriate. Accordingly, the evidence was synthesized narratively.

Information Sources and Search Strategy

A comprehensive literature search was conducted in PubMed/MEDLINE, Embase, and Scopus. Google Scholar was used as a supplementary source to identify potentially relevant publications not retrieved through the primary database searches. The search covered studies published from January 1990 through May 2026, thereby encompassing the modern era of laparoscopic cholecystectomy and contemporary approaches to the prevention, diagnosis, and management of bile duct injury. The final search was conducted on May 10, 2026.

Search strategies combined controlled vocabulary, including Medical Subject Headings and database-specific indexing terms where applicable, with free-text terms related to cholecystectomy, bile duct injury, prevention, diagnosis, and management. Boolean operators were used to combine the principal concepts. The complete search strategies for each database are provided in** **Table 1.

**Table 1:** Electronic search strategies used across databases Searches covered publications from January 1990 through May 10, 2026. Google Scholar was used as a supplementary source. MeSH, Medical Subject Headings; ERCP, endoscopic retrograde cholangiopancreatography; MRCP, magnetic resonance cholangiopancreatography.

Database	Search strategy
PubMed/MEDLINE	(("Cholecystectomy/adverse effects"[MeSH Terms] OR "Cholecystectomy"[MeSH Terms] OR cholecystectomy[Title/Abstract] OR "laparoscopic cholecystectomy"[Title/Abstract] OR postcholecystectomy[Title/Abstract] OR "post-cholecystectomy"[Title/Abstract]) AND ("Bile Ducts/injuries"[MeSH Terms] OR "Biliary Tract/injuries"[MeSH Terms] OR "bile duct injury"[Title/Abstract] OR "bile duct injuries"[Title/Abstract] OR "biliary injury"[Title/Abstract] OR "biliary tract injury"[Title/Abstract] OR "iatrogenic bile duct injury"[Title/Abstract] OR "bile leak"[Title/Abstract] OR "biliary stricture"[Title/Abstract]) AND (prevention[Title/Abstract] OR diagnosis[Title/Abstract] OR diagnostic[Title/Abstract] OR management[Title/Abstract] OR treatment[Title/Abstract] OR therapy[Title/Abstract] OR repair[Title/Abstract] OR reconstruction[Title/Abstract] OR hepaticojejunostomy[Title/Abstract] OR cholangiography[Title/Abstract] OR ERCP[Title/Abstract] OR MRCP[Title/Abstract] OR drainage[Title/Abstract])) AND ("1990/01/01"[Date - Publication] : "2026/05/10"[Date - Publication])
Embase	('cholecystectomy'/exp OR 'laparoscopic cholecystectomy'/exp OR cholecystectomy:ti,ab OR 'laparoscopic cholecystectomy':ti,ab OR postcholecystectomy:ti,ab OR 'post-cholecystectomy':ti,ab) AND ('bile duct injury'/exp OR 'biliary tract injury'/exp OR 'bile duct injury':ti,ab OR 'bile duct injuries':ti,ab OR 'biliary injury':ti,ab OR 'biliary tract injury':ti,ab OR 'iatrogenic bile duct injury':ti,ab OR 'bile leak':ti,ab OR 'biliary stricture':ti,ab) AND ('prevention'/exp OR prevention:ti,ab OR 'diagnosis'/exp OR diagnosis:ti,ab OR diagnostic:ti,ab OR 'therapy'/exp OR management:ti,ab OR treatment:ti,ab OR therapy:ti,ab OR repair:ti,ab OR reconstruction:ti,ab OR hepaticojejunostomy:ti,ab OR 'endoscopic retrograde cholangiopancreatography'/exp OR ERCP:ti,ab OR MRCP:ti,ab OR 'intraoperative cholangiography':ti,ab OR stent:ti,ab OR drainage:ti,ab) AND (1990-2026)/py
Scopus	TITLE-ABS-KEY(cholecystectomy OR "laparoscopic cholecystectomy" OR postcholecystectomy OR "post-cholecystectomy") AND TITLE-ABS-KEY("bile duct injury" OR "bile duct injuries" OR "biliary tract injury" OR "biliary tract injuries" OR "biliary injury" OR "biliary injuries" OR "iatrogenic bile duct injury" OR "bile leak" OR "biliary stricture" OR "major bile duct injury") AND TITLE-ABS-KEY(prevention OR diagnosis OR diagnostic OR management OR treatment OR therapy OR repair OR reconstruction OR hepaticojejunostomy OR ERCP OR "endoscopic retrograde cholangiopancreatography" OR MRCP OR cholangiography OR "percutaneous drainage" OR "Strasberg classification") AND PUBYEAR > 1989 AND PUBYEAR < 2027
Google Scholar	("bile duct injury" OR "biliary injury" OR "iatrogenic bile duct injury" OR "bile leak") AND ("laparoscopic cholecystectomy" OR cholecystectomy OR postcholecystectomy) AND (prevention OR diagnosis OR management OR treatment OR repair OR reconstruction)

Equivalent strategies were adapted to the indexing structure and syntax of Embase and Scopus. Google Scholar was searched using simplified combinations of the same core concepts, and the first 20 results ranked by relevance were screened. The reference lists of eligible primary studies, clinical practice guidelines, consensus statements, and selected reviews were also examined to identify additional potentially eligible primary studies.

Grey literature, conference abstracts, posters, unpublished reports, preprints, and other non-peer-reviewed materials were excluded. Only publications for which the full text was successfully retrieved were considered for eligibility assessment. 

Eligibility Criteria

Studies were eligible if they evaluated adult patients with iatrogenic bile duct injury associated with laparoscopic, open, or converted cholecystectomy and addressed at least one of the following domains: prevention or intraoperative recognition; diagnostic evaluation or injury classification; endoscopic, radiological, surgical, or multidisciplinary management; timing of referral or definitive reconstruction; healthcare system, institutional, or referral-related factors; or short- and long-term clinical outcomes.

To be included as primary evidence, studies were required to report at least one clinically relevant outcome, including the incidence of bile duct injury, timing of diagnosis, bile leak resolution, treatment success, postoperative complications, biliary stricture, need for reintervention, mortality, quality of life, healthcare utilization, or economic burden.

Eligible primary study designs included prospective and retrospective cohort studies, comparative and noncomparative observational studies, population-based analyses, single-center or multicenter institutional series, and case series involving more than five patients. Clinical practice guidelines, consensus statements, and selected narrative reviews were consulted to support the interpretation of the evidence, provide historical or technical context, and identify potentially relevant primary publications. These sources were not considered primary evidence and were not included in the narrative synthesis or the final PRISMA count.

Studies published in English, Spanish, or Portuguese between January 1990 and May 2026 were eligible.

Exclusion Criteria

Studies were excluded if they focused on pediatric populations; evaluated noniatrogenic bile duct or biliary tract injuries; addressed cholecystectomy without reporting BDI-specific findings; consisted exclusively of animal, cadaveric, in vitro, or computational investigations; described isolated technical modifications without clinically interpretable outcomes; included five or fewer patients; were editorials, letters, opinion pieces, commentaries, protocols without results, conference abstracts, or posters; or did not provide sufficient clinical information relevant to the objectives of the review.

Systematic reviews and meta-analyses were not included as primary evidence or in the final PRISMA count but were consulted to identify potentially relevant studies and support the contextual interpretation of the findings. Duplicate publications, overlapping cohorts, and secondary analyses of previously reported populations were assessed to minimize double counting. When substantial cohort overlap was identified, the most comprehensive, methodologically robust, or clinically informative publication was prioritized.

Study Selection

All records retrieved through the electronic database searches were exported to a structured Microsoft Excel (Microsoft Corporation, Redmond, USA) spreadsheet for organization and screening. Duplicate records were manually identified and removed before title and abstract screening. Potentially relevant publications were subsequently retrieved and assessed in full text according to the predefined eligibility criteria. Reasons for exclusion at the full-text stage were documented using standardized categories. Study eligibility was assessed independently by two reviewers, with disagreements resolved through discussion and consensus.

A total of 190 records were identified through searches of PubMed/MEDLINE, Embase, Scopus, and Google Scholar. After the removal of eight duplicate records, 182 unique records underwent title and abstract screening. Of these, 124 were excluded, and 58 publications were retrieved and assessed in full text.

**Figure 1:**
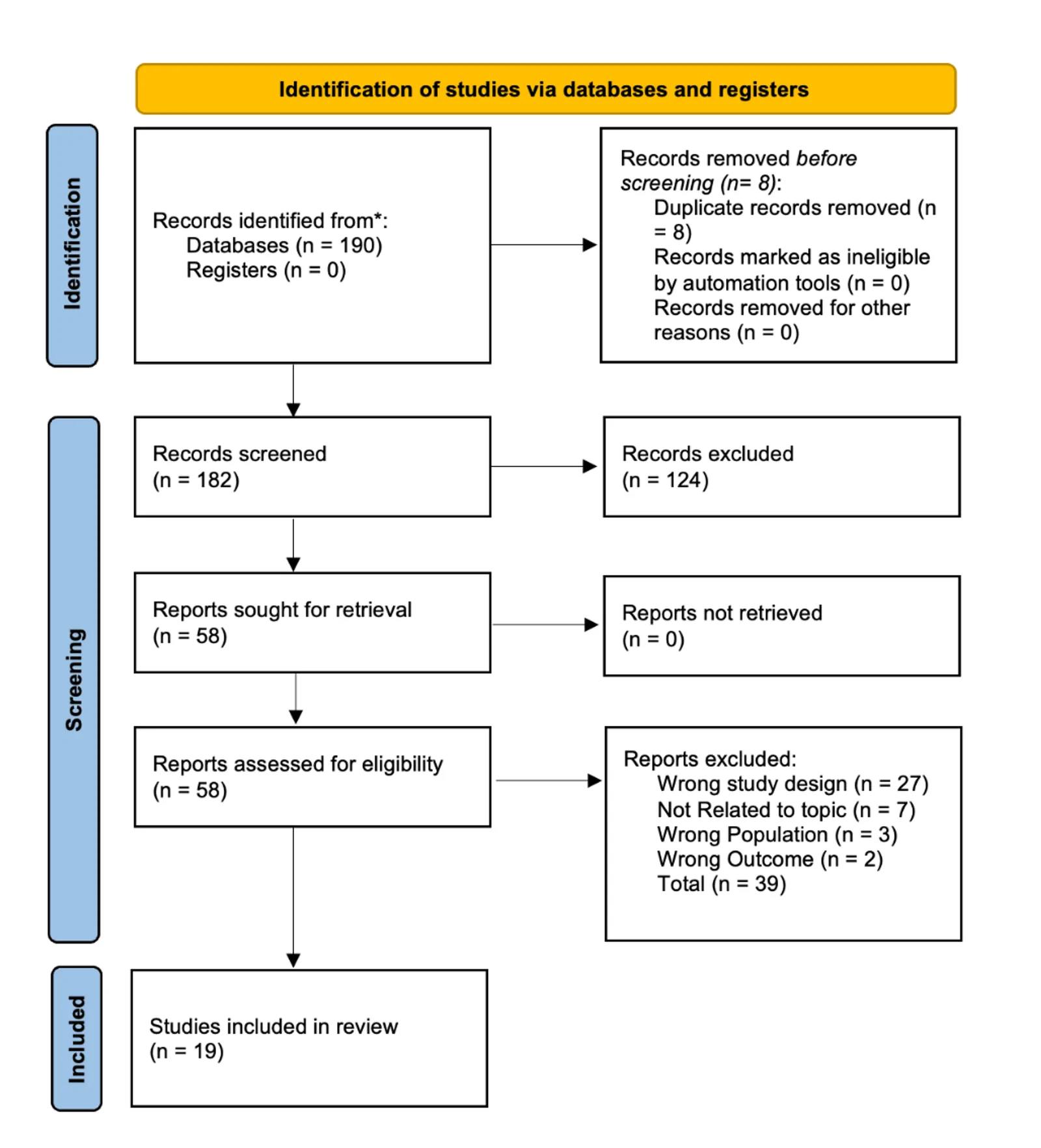
PRISMA 2020 flow diagram of study identification, screening, eligibility assessment, and inclusion. Flow diagram illustrating the identification, screening, eligibility assessment, and final inclusion of studies in this structured narrative review. A total of 190 records were identified through database searches, of which 19 primary studies met the predefined eligibility criteria and were included in the final narrative synthesis. PRISMA: Preferred Reporting Items for Systematic Reviews and Meta-Analyses

Thirty-nine full-text publications were excluded because of an ineligible study design (n = 27), lack of relevance to the review question (n = 7), an ineligible study population (n = 3), or absence of clinically relevant outcomes (n = 2). Ultimately, 19 primary studies met the eligibility criteria and were included in the narrative synthesis. One narrative review was excluded from the primary-study synthesis because of its study design but was subsequently consulted for contextual and interpretative purposes. The complete study selection process is presented in the PRISMA 2020 flow diagram (Figure 1). 

Data Extraction

Data were extracted using a standardized Microsoft Excel spreadsheet developed before evidence synthesis. For each eligible primary study, relevant information was recorded using predefined data fields to promote consistency across the extraction process. Data extraction was performed independently by two authors, and any discrepancies were resolved by a third author.

The following variables were collected: authors, year of publication, country of origin, study design, healthcare setting, patient characteristics, sample size, type and classification of bile duct injury, diagnostic approach, treatment strategy, timing of referral or intervention, duration of follow-up, and principal clinical outcomes. Outcomes of interest included bile leak resolution, postoperative complications, biliary stricture, treatment failure, need for reintervention, morbidity, mortality, quality of life, and other long-term clinical outcomes, when reported.

Additional methodological characteristics, including the presence of a comparison group, completeness of follow-up, consistency of outcome definitions, and adequacy of outcome reporting, were documented to support interpretation of the available evidence. Missing, unclear, or unavailable information was recorded as not reported and was not inferred. The completed dataset served as the basis for the subsequent narrative synthesis.

Quality Assessment

Given the structured narrative design of this review and the substantial heterogeneity anticipated across the included studies in terms of study design, patient populations, interventions, healthcare settings, follow-up, and outcome reporting, a formal risk-of-bias assessment using a standardized critical appraisal tool was not performed.

Instead, methodological characteristics relevant to the interpretation of the evidence were systematically extracted and considered during the narrative synthesis. These included study design, sample size, presence of a comparison group, healthcare setting, duration and completeness of follow-up, consistency of outcome definitions, and adequacy of outcome reporting.

This information was used descriptively to contextualize the strength, applicability, and limitations of the available evidence. Studies were not excluded solely on the basis of methodological limitations, and no numerical quality score or overall certainty rating was assigned.

Data Synthesis

Due to the clinical and methodological heterogeneity of the included studies in terms of study design, patient populations, injury characteristics and severity, diagnostic approaches, therapeutic strategies, healthcare settings, and reported outcomes, the evidence was synthesized narratively.

Studies were organized according to predefined thematic domains relevant to the review objectives, including prevention and intraoperative recognition of bile duct injury; diagnostic evaluation and injury classification; endoscopic, radiological, surgical, and multidisciplinary management; timing of referral and definitive reconstruction; clinical outcomes and complications; and healthcare system challenges relevant to the Mexican context.

Within each domain, findings were descriptively summarized and compared according to study design, patient population, injury pattern, management strategy, healthcare setting, and reported outcomes. Areas of consistency, clinical variation, and disagreement across the literature were identified while preserving the methodological context of individual studies.

Because of substantial heterogeneity in study designs, interventions, patient populations, and outcome definitions, statistical pooling was not considered appropriate, and no meta-analysis was performed.

Narrative synthesis

Nineteen primary studies fulfilled the predefined eligibility criteria and were included in the narrative synthesis. The evidence base consisted predominantly of observational studies, including population-based, multicenter, and institutional cohorts, supplemented by cross-sectional studies and case series. Collectively, these studies represented diverse healthcare settings across North America, Latin America, Europe, and Asia and addressed the continuum of post-cholecystectomy bile duct injury, including prevention, intraoperative recognition, referral, definitive reconstruction, healthcare-system organization, and long-term outcomes. The methodological characteristics and principal contributions of the included primary studies are summarized in Table 2 [2-20]. One additional narrative review was consulted exclusively for contextual and interpretative support and was not considered primary evidence or included in the narrative synthesis. 

**Table 2:** Characteristics of studies contributing to the narrative synthesis BDI, bile duct injury; CVS, Critical View of Safety.

Study	Country/region	Study design	Sample size	Main clinical domain	Principal contribution
Chávez-Villa et al. (2021) [2]	México	Comparative cohort	408	Bailout strategies	Compared subtotal cholecystectomy after failure to achieve CVS with patients sustaining established BDI.
Cuendis-Velázquez et al. (2019) [3]	México	Comparative cohort	75	Minimally invasive reconstruction	Compared laparoscopic and robotic hepaticojejunostomy for major BDI.
Rueda-De-León et al. (2020) [4]	México and United States	Comparative referral-center cohort	383	Referral disparities	Compared referral timing, previous repairs, reconstructive complexity, and biliary patency between healthcare settings.
Mercado et al. (2008) [5]	México	Comparative institutional cohort	405	Surgical reconstruction	Evaluated intrahepatic versus extrahepatic approaches for reconstruction of major BDI.
Christou et al. (2021) [6]	France	Retrospective cross-sectional study	22	Intraoperative recognition	Evaluated mechanisms of injury and the performance and interpretation of intraoperative cholangiography.
Kalata et al. (2023) [7]	United States	Population-based retrospective comparative cohort	1,026,088	Surgical platform and bile duct injury risk	Compared robotic-assisted versus laparoscopic cholecystectomy. Robotic procedures were associated with a higher frequency of major BDI requiring biliary reconstruction (0.7% vs. 0.2%) and minor postoperative biliary interventions (7.4% vs. 6.0%), despite similar overall 30-day complication rates.
Mullens et al. (2025) [8]	United States	Population-based comparative cohort	737,908	Robotic cholecystectomy and BDI	Examined whether patient complexity explained differences in BDI requiring operative intervention between surgical platforms.
Martínez-Mier et al. (2020) [9]	México	Comparative cohort	78	Healthcare-system variation	Compared BDI presentation, referral, management, and outcomes between two Mexican public institutions.
Cuendis-Velázquez et al. (2018) [10]	México	Case series	30	Robotic reconstruction	Assessed feasibility and early outcomes of robotic Roux-en-Y hepaticojejunostomy after BDI.
Conde-Monroy et al. (2022) [11]	Colombia	Multicenter retrospective cohort	44	Timing of reconstruction	Compared early and delayed reconstruction after major BDI across hepatopancreatobiliary centers.
Chartkitchareon and Tullavardhana (2025) [12]	Thailand	Retrospective cross-sectional study	50	Prevention and intraoperative imaging	Examined integration of the CVS with fluorescence cholangiography.
Zhang et al. (2024) [13]	China	Prospective comparative study	276	Intraoperative prevention	Evaluated cystic duct exploration as an adjunct during laparoscopic cholecystectomy.
Yang et al. (2024) [14]	China	Case series	15	Surgical planning	Assessed three-dimensional visualization for anatomical characterization and early BDI repair.
Koppatz et al. (2020) [15]	Finland	Long-term cohort study	52	Quality of life and reconstruction	Evaluated long-term survival, biliary complications, reinterventions, and quality of life after major BDI.
Abou Assali et al. (2025) [16]	United States	Multicenter observational cohort	93,122	Robotic cholecystectomy	Compared perioperative outcomes of robotic and laparoscopic elective cholecystectomy.
Giuliante et al. (2023) [17]	Italy	Retrospective cohort	243 admitted; 114 reconstructed	Timing and surgical reconstruction	Examined timing of repair and long-term outcomes after Hepp–Couinaud hepaticojejunostomy.
Jayasekara et al. (2024) [18]	Sri Lanka	Cohort study	19	Long-term outcomes	Assessed liver stiffness, anastomotic stenosis, recurrent cholangitis, and long-term outcomes after major BDI reconstruction.
Losada et al. (2022) [19]	Chile	Cohort study	32	Quality of life	Evaluated long-term clinical outcomes and quality of life following biliary reconstruction.
Ng et al. (2025) [20]	United States	National retrospective cohort	152,687	Intraoperative cholangiography	Evaluated utilization of intraoperative cholangiography, conversion, readmission, and resource use.

Incidence, Underreporting, and Healthcare-System Gaps in Post-cholecystectomy Bile Duct Injury in Mexico

The epidemiology of post-cholecystectomy bile duct injury in Mexico is supported by a limited and predominantly institutional evidence base. International studies have reported incidence rates ranging from approximately 0.2% to 0.9% for major injuries and up to 1.5%-1.8% when minor lesions are included [1,6]. Several contemporary estimates have been derived from large administrative databases, including the U.S. Medicare system [7,8]. In contrast, Mexico lacks a national registry or population-based surveillance system dedicated to bile duct injury. Incidence estimates most frequently cited in the Mexican literature, generally ranging from 0.2% to 0.6%, are therefore largely extrapolated from international series rather than calculated from national population-based data [9].

The Mexican studies identified in this review were primarily institutional cohorts and case series from tertiary referral centers, particularly the Instituto Nacional de Ciencias Médicas y Nutrición Salvador Zubirán and the Dr. Manuel Gea González General Hospital [2,3,5,10]. These reports characterize patients referred for specialist treatment but do not provide a national denominator based on the total number of cholecystectomies performed. Although other Mexican centers may have published selected experiences with bile duct injury, no additional national studies meeting the predefined eligibility criteria were identified through the present search strategy. Consequently, the true national incidence remains uncertain and cannot be directly estimated from the available evidence.

Potential underreporting may further limit case ascertainment across the spectrum of injury severity. Minor injuries managed conservatively outside specialist referral networks may not be captured, whereas patients who die before referral or definitive reconstruction may also be absent from institutional cohorts. A Colombian multicenter study similarly acknowledged limitations in case capture and reported an institutional incidence of 0.07% [11]. Although direct Mexican evidence is lacking, comparable gaps in surveillance are plausible. Concomitant vasculobiliary injuries have also been described as incompletely documented [9]. In a cross-national referral-center comparison, only 0.6% of Mexican patients were referred within three days of injury, compared with 51.6% in the U.S. cohort, and more than half of the Mexican patients were referred more than six weeks after the index injury [4]. These findings demonstrate substantial delays in referral, although their effect on national case registration cannot be quantified from the available data.

Differences in operative approach further complicate comparisons across healthcare systems. Large U.S. database studies have associated robotic-assisted cholecystectomy with higher rates of bile duct injury requiring operative intervention than conventional laparoscopic cholecystectomy [7,8]. Mexican referral cohorts, however, show a markedly different operative profile. Open cholecystectomy accounted for 55.4% of index procedures preceding referred injuries, compared with 3.3% in the U.S. comparator cohort [4]. This difference may reflect variation in access to minimally invasive surgery, institutional resources, surgeon experience, case complexity, and referral patterns. Considerable variation has also been reported within the Mexican public healthcare system, with open cholecystectomy accounting for 48.6% of index procedures in the Instituto Mexicano del Seguro Social and 73% in the Servicios de Salud de Veracruz [9].

Cross-national comparisons also suggest differences in access to definitive specialist care. Mexican patients had lower primary biliary patency than their U.S. counterparts (83% vs. 93%) and a higher proportion of unsuccessful repair attempts before referral (42.9%) [4]. These findings suggest that delayed referral, limited access to hepatopancreatobiliary expertise, and previous nonspecialist repair may contribute to greater reconstructive complexity and poorer long-term outcomes. However, these observations arise from selected referral cohorts and should not be interpreted as nationally representative estimates.

No Mexican population-based data were identified regarding the incidence of bile duct injury associated with robotic-assisted cholecystectomy. Published Mexican experience with robotic surgery has focused primarily on biliary reconstruction rather than the index cholecystectomy [3,10]. Accordingly, the safety profile of robotic cholecystectomy in Mexico remains insufficiently characterized.

Overall, the available evidence suggests that the epidemiology of bile duct injury in Mexico is characterized by incomplete surveillance, delayed referral, concentration of reported cases in tertiary centers, and substantial variation in access to minimally invasive and specialist hepatobiliary care. Whether these disparities are primarily attributable to differences between public and private healthcare settings or to broader structural limitations across the health system cannot be determined from the current literature.

Biliary Anatomical Variants and Patient-Related Factors Influencing Operative Risk

Anatomical variability is an important contributor to operative difficulty during cholecystectomy and may increase the risk of BDI. The cystic artery follows an atypical course in approximately 25% of patients, whereas accessory bile ducts, including aberrant right sectoral ducts, have been reported in 1-30% of cases [6]. Injury to ducts that do not communicate with the main biliary tree may result in postoperative bile leakage that is not visualized on conventional cholangiography. Low-lying or atypical biliary confluences may also complicate both operative dissection and subsequent biliary reconstruction when injured [5]. Human-factors analyses further suggest that many BDIs result from visual misperception and anatomical misidentification rather than an isolated technical error, particularly when the common bile duct is mistaken for the cystic duct [6].

The clinical relevance of anatomical variation becomes greatest when safe identification of biliary structures cannot be achieved. Failure to obtain the Critical View of Safety (CVS) has been associated with inflammatory changes, previous endoscopic retrograde cholangiopancreatography, longer operative times, and more frequent use of subtotal cholecystectomy [12]. Intraoperative cholangiography may assist in defining uncertain biliary anatomy and recognizing injury, although its preventive effectiveness remains inconsistent and depends on correct acquisition and interpretation [6]. Other adjuncts, including indocyanine green fluorescence cholangiography and cystic duct exploration, may improve anatomical visualization in selected patients but should be regarded as complementary to, rather than substitutes for, meticulous dissection and reliable anatomical identification [12,13].

Patient-related and local pathological factors may further increase operative complexity. Acute or chronic inflammation, fibrosis, and involvement of the hilar region are frequently reported in patients managed for BDI [6]. Obesity, greater comorbidity burden, and higher American Society of Anesthesiologists physical status may identify patients with more complex operative conditions, although the available evidence does not establish these characteristics as independent causal determinants of injury [2]. Previous abdominal surgery and adhesions may similarly restrict exposure and complicate dissection [2,6].

Evidence from a Mexican cohort suggests that subtotal cholecystectomy may provide a safer alternative when difficult operative conditions preclude secure anatomical identification. Patients undergoing subtotal cholecystectomy had a greater burden of comorbidity, acute cholecystitis, and emergency surgery than patients who sustained BDI, yet experienced fewer reoperations and no cases of secondary biliary cirrhosis during follow-up [2]. These findings support the use of bailout strategies in selected difficult cholecystectomies, although they derive from observational data and should not be interpreted as definitive comparative evidence. Conversely, BDIs may also occur during elective procedures without marked inflammation, and population-based studies have reported elevated injury rates across different preoperative risk strata [7,8]. Patient characteristics alone therefore appear insufficient to reliably predict injury occurrence.

Preoperative evaluation may be more useful for anticipating operative difficulty than for predicting BDI directly. Selective magnetic resonance cholangiopancreatography has not been validated as a routine screening strategy for injury prevention, although careful review of preoperative imaging and previous operative reports may support planning in complex cases [1,14]. When multiple adverse anatomical or clinical factors are present, the available evidence supports consideration of early bailout procedures, intraoperative consultation, or referral to hepatopancreatobiliary specialists, particularly when safe biliary identification cannot be achieved [4,15].

High-Risk Operative Conditions, Bailout Strategies, and Prevention of Bile Duct Injury

Prevention of BDI depends largely on recognizing when conventional dissection can no longer be performed safely. Human-factors analyses suggest that many major injuries result from misinterpretation of distorted biliary anatomy rather than from an isolated technical error [6]. Acute cholecystitis, dense inflammation within the hepatocystic triangle, severe fibrosis, a contracted gallbladder, uncontrolled bleeding, and poorly defined or aberrant biliary anatomy may impair spatial orientation and increase the likelihood of anatomical misidentification [2,6,12]. Under these conditions, inability to establish a reliable anatomical view should prompt reassessment and modification of the operative strategy rather than persistence with increasingly hazardous dissection.

The CVS remains the principal method for confirming biliary anatomy before clipping or dividing ductal structures. Its importance extends beyond anatomical identification, because failure to achieve CVS should function as a decision point for transition to an alternative operative strategy. Available options include fundus-first dissection, subtotal cholecystectomy, selective intraoperative cholangiography (IOC), indocyanine green (ICG) fluorescence cholangiography, temporary drainage in selected cases, and conversion to an open approach when adequate exposure, anatomical definition, or vascular control cannot be achieved [2,6,12].

Intraoperative cholangiography may facilitate clarification of uncertain biliary anatomy and intraoperative recognition of injury, although its preventive effectiveness remains inconsistent and depends on correct performance and interpretation [6]. Similarly, ICG fluorescence cholangiography provides real-time visualization of the extrahepatic biliary tree and may improve anatomical orientation in selected cases, but the available evidence is predominantly observational and does not support its use as a substitute for CVS [12].

Among bailout procedures, subtotal cholecystectomy has demonstrated favorable safety outcomes in difficult operative settings. In one Mexican cohort, postoperative bile leakage occurred in 21% of patients undergoing subtotal cholecystectomy, whereas major BDI occurred in 0.9% [2]. Patients who sustained an established BDI subsequently required more reoperations and experienced secondary biliary cirrhosis more frequently during follow-up. Although these findings derive from observational data and do not establish definitive comparative effectiveness, they support subtotal cholecystectomy as a reasonable alternative when safe completion of a total cholecystectomy is not feasible.

Technological advances may improve visualization and operative ergonomics, but they do not replace sound anatomical interpretation or structured intraoperative decision-making. Evidence regarding robotic-assisted cholecystectomy remains conflicting. Two large Medicare-based cohort studies reported an approximately threefold higher rate of BDI requiring operative intervention after robotic-assisted compared with conventional laparoscopic cholecystectomy, and this association persisted across different preoperative risk strata [7,8]. In contrast, the multicenter ROBOCOP study reported lower conversion rates and fewer coded BDIs with the robotic approach [16]. These divergent findings may reflect differences in patient selection, surgeon experience, procedural volume, institutional practice, outcome coding, and study design rather than an effect attributable solely to the surgical platform.

Overall, the available evidence favors prevention strategies based on standardized training, competency-based adoption of new technologies, reliable assessment of CVS, predefined bailout algorithms, selective use of intraoperative imaging, and timely access to hepatopancreatobiliary expertise [4,15]. Subtotal cholecystectomy, conversion, temporary drainage, and referral should therefore be regarded as safety-oriented responses to difficult operative conditions rather than as indicators of technical failure. Referral studies have also shown higher primary biliary patency after initial repair by experienced hepatopancreatobiliary surgeons, whereas previous unsuccessful repair attempts are associated with more complex reconstruction and poorer long-term outcomes [4,15]. Table 3 summarizes the principal intraoperative risk factors and corresponding mitigation strategies identified in the literature.

**Table 3:** Principal risk factors and mitigation strategies for bile duct injury Principal patient-related, anatomical, technical, procedural, technological, and healthcare-system factors associated with bile duct injury during cholecystectomy, together with their reported clinical implications and corresponding mitigation strategies. BDI, bile duct injury; CVS, Critical View of Safety; IOC, intraoperative cholangiography; ICG, indocyanine green

Domain	Associated factor	Reported impact	Mitigation strategy	References
Patient and inflammatory factors	Acute cholecystitis and severe gallbladder or biliary inflammation	Inflammatory distortion of the hepatocystic triangle increases operative difficulty, limits achievement of CVS, and favors anatomical misidentification.	Anticipate a difficult cholecystectomy; repeatedly reassess anatomical orientation; use adjunctive imaging selectively; and adopt an early bailout strategy when CVS cannot be safely achieved.	[2,12]
Biliary anatomy	Aberrant, distorted, or poorly defined biliary anatomy	Misidentification of the cystic duct, common bile duct, or adjacent vascular structures is a major mechanism of BDI, particularly in the presence of inflammation or difficult dissection.	Achieve CVS before clipping or transection; avoid division of unidentified structures; and use intraoperative biliary imaging when anatomy remains uncertain.	[2,6,12]
Surgeon and technical factors	Limited experience, inadequate anatomical interpretation, and platform-specific learning curves	Failure to recognize or correctly interpret biliary anatomy may delay intraoperative diagnosis and increase the risk of major injury.	Provide structured training in biliary anatomy, CVS assessment, and interpretation of intraoperative imaging; use supervision or proctoring during adoption of new surgical platforms.	[6–8]
Operative strategy	Failure to achieve CVS	Continued dissection in an unsafe hepatocystic triangle increases the likelihood of anatomical misidentification and major BDI.	Stop unsafe dissection and transition promptly to a predefined bailout strategy rather than pursuing total cholecystectomy at all costs.	[2,12]
Bailout procedure	Hostile operative field or persistent inability to define biliary anatomy safely	Subtotal cholecystectomy carries a recognized risk of postoperative bile leakage but may avoid the greater long-term morbidity associated with major BDI.	Perform subtotal cholecystectomy, fundus-first dissection, drainage, or conversion according to operative findings and available expertise.	[2,12]
Intraoperative imaging	Uncertain anatomy or suspected injury	IOC may facilitate earlier recognition of BDI, whereas ICG fluorescence may improve real-time visualization of extrahepatic biliary anatomy. Their effectiveness depends on appropriate use and interpretation.	Use IOC selectively when anatomy or injury is uncertain; ensure competency in image interpretation; consider ICG fluorescence as an adjunct rather than a replacement for CVS.	[6,12]
Surgical platform	Robotic-assisted cholecystectomy and platform-specific learning curve	Large population-based studies have reported higher rates of BDI requiring operative intervention after robotic-assisted cholecystectomy, although findings remain inconsistent across studies.	Ensure structured robotic training, appropriate case selection, supervision, and sufficient procedural experience before independent adoption.	[7,8,16]
Healthcare system	Delayed referral, nonspecialist repair, and limited access to experienced hepatopancreatobiliary care	Previous repair attempts and delayed referral are associated with more complex reconstruction and lower long-term biliary patency.	Refer major or complex injuries early to specialized centers and avoid definitive repair when appropriate hepatopancreatobiliary expertise is unavailable.	[4,15]

Diagnostic and Therapeutic Pathways for Post-cholecystectomy Bile Duct Injury

Successful management of post-cholecystectomy BDI requires early recognition and a structured pathway integrating clinical stabilization, cross-sectional imaging, endoscopy, interventional radiology, and definitive surgical reconstruction when indicated. Clinical presentation is variable and may include failure to recover as expected, abdominal pain, fever, external bile drainage, jaundice, cholangitis, biliary peritonitis, or sepsis. Because no single diagnostic modality is appropriate for every injury pattern, evaluation should be guided by the patient’s clinical stability, suspected ductal continuity, presence of intra-abdominal contamination, and likelihood of associated vascular injury [1].

In hemodynamically stable patients with suspected postoperative BDI, contrast-enhanced abdominal computed tomography (CT) is an appropriate initial imaging modality, particularly when intra-abdominal collections, biloma, biliary dilatation, hepatic hypoperfusion, or vascular injury are suspected. A multiphase protocol including an arterial phase may improve assessment of concomitant hepatic arterial injury. Transabdominal ultrasonography may identify fluid collections or intrahepatic ductal dilatation but generally provides less comprehensive anatomical information. Once urgent complications have been assessed, magnetic resonance cholangiopancreatography (MRCP), with hepatobiliary contrast enhancement when available and clinically appropriate, can provide noninvasive characterization of the biliary tree, level of obstruction, proximal ductal anatomy, and probable site of bile leakage [1].

Endoscopic retrograde cholangiopancreatography (ERCP) has both diagnostic and therapeutic roles but should be selected according to the injury pattern and preservation of ductal continuity. It is most useful for cystic duct stump leaks, accessory duct leaks communicating with the biliary tree, and minor lateral injuries in which continuity of the main bile duct is maintained. ERCP may demonstrate the site and severity of leakage, identify retained stones, and reduce the transpapillary pressure gradient through sphincterotomy or stent placement. Clinical resolution has been reported in more than 90% of appropriately selected postoperative bile leaks [1].

The role of ERCP is more limited in complete transection, disconnected proximal ducts, and complex hilar injuries, because the proximal biliary system may not be accessible from the duodenum. In these settings, percutaneous transhepatic cholangiography and drainage may be required to define or decompress the proximal ducts, while definitive management commonly involves surgical reconstruction. In selected complex injuries, three-dimensional reconstruction based on CT or magnetic resonance imaging may further clarify the relationship between multiple ductal openings and adjacent hilar vessels, although the supporting evidence remains limited [1,14].

In patients with biloma, biliary peritonitis, cholangitis, or abdominal sepsis, source control and physiological stabilization should precede definitive reconstruction. Image-guided percutaneous drainage, biliary decompression when required, and antimicrobial therapy are central components of initial management. Operative lavage and drainage may be necessary when percutaneous intervention is unavailable, technically infeasible, or insufficient to control contamination. Definitive repair should generally be deferred until the injury pattern, biliary anatomy, inflammatory status, and presence of associated vascular injury have been adequately characterized [1,11].

When a major injury is recognized intraoperatively, immediate consultation with an experienced hepatopancreatobiliary surgeon is preferable. Intraoperative cholangiography may help define ductal continuity, the level of injury, and the relationship to the biliary confluence when technically feasible. If appropriate expertise is unavailable, placement of drains, avoidance of an unplanned definitive reconstruction, and prompt referral to a specialized center may reduce the complexity of subsequent repair [6,11]. Table 4 summarizes the diagnostic and initial therapeutic pathway according to clinical presentation and suspected injury pattern.

**Table 4:** Diagnostic and initial therapeutic approach according to clinical presentation Diagnostic and initial therapeutic approach to suspected post-cholecystectomy bile duct injury according to clinical presentation and probable injury pattern. CT, computed tomography; ERCP, endoscopic retrograde cholangiopancreatography; MRCP, magnetic resonance cholangiopancreatography

Clinical presentation	Principal diagnostic concern	Initial evaluation	Complementary evaluation or intervention	Clinical role	References
Failure to recover after cholecystectomy, abdominal pain, fever, or nonspecific postoperative deterioration	Occult bile leak, biliary obstruction, intra-abdominal collection, or associated vascular injury	Contrast-enhanced abdominal CT; consider a multiphase protocol with an arterial phase	MRCP, with hepatobiliary contrast enhancement when appropriate	CT identifies collections, biloma, biliary dilatation, and vascular or perfusion abnormalities; MRCP further characterizes the biliary anatomy and probable injury site.	[1]
External bile drainage or suspected postoperative bile leak	Cystic duct stump leak, accessory duct leak, or partial injury with preserved ductal continuity	CT or ultrasonography to assess associated collections	MRCP for anatomical characterization; ERCP when ductal continuity is preserved and endoscopic treatment is feasible	Cross-sectional imaging evaluates associated contamination, whereas ERCP confirms the leak and permits decompression or stent placement.	[1,14]
Obstructive jaundice or cholestatic biochemical abnormalities	Bile duct clipping, ligation, transection, or postoperative stricture	Contrast-enhanced CT	MRCP; percutaneous transhepatic cholangiography when proximal ducts cannot be adequately accessed or characterized	CT evaluates ductal dilatation, liver perfusion, and vascular complications; MRCP defines the level and extent of obstruction and proximal ductal anatomy.	[1,14]
Biloma, biliary peritonitis, cholangitis, or abdominal sepsis	Active bile leak with infected or undrained contamination	Urgent contrast-enhanced CT	Image-guided percutaneous drainage, antimicrobial therapy, and subsequent MRCP or ERCP when clinically appropriate	Source control and stabilization take priority over definitive endoscopic or surgical reconstruction.	[1,11]
Injury recognized or suspected intraoperatively	Partial or complete major BDI, clipping, transection, or associated vascular injury	Direct operative assessment and intraoperative cholangiography when feasible	Immediate hepatopancreatobiliary consultation; postoperative CT or MRCP when anatomy or vascular status remains uncertain	Intraoperative imaging may define ductal continuity and injury level. When specialist expertise is unavailable, drainage and prompt referral are preferable to unplanned definitive repair.	[6,11]
Complete transection or disconnected proximal ducts	Loss of continuity between the proximal and distal biliary systems	Multiphase CT followed by MRCP	Percutaneous transhepatic cholangiography and drainage; specialist evaluation for surgical reconstruction	ERCP may not opacify or access the proximal ducts; percutaneous access can define anatomy and provide decompression before reconstruction.	[1,14]
Complex proximal, hilar, or vasculobiliary injury	High-grade Strasberg E injury, separated hilar ducts, multiple biliary openings, or associated arterial injury	Multiphase CT and MRCP	Percutaneous cholangiography and selective three-dimensional imaging reconstruction for operative planning	Multimodal imaging defines the proximal biliary anatomy, vascular involvement, and number and location of ductal openings before definitive repair.	[1,14]

Referral Pathways, Centralization, and Regional Treatment Capacity

Management of post-cholecystectomy BDI depends not only on surgical expertise but also on timely access to specialized diagnostic and therapeutic resources. These include endoscopic retrograde cholangiopancreatography, magnetic resonance cholangiopancreatography, contrast-enhanced computed tomography, intraoperative cholangiography, interventional radiology, and hepatopancreatobiliary (HPB) surgery. Across healthcare systems, access to these resources remains heterogeneous and may influence the timing of diagnosis, source control, referral, and definitive reconstruction [1,17].

Available Mexican reports suggest that advanced biliary imaging, therapeutic endoscopy, interventional radiology, and specialized HPB care are concentrated predominantly in high-complexity urban referral centers [4,9,11]. However, the current literature does not provide a comprehensive national assessment of resource availability across tertiary, regional, public, and private institutions. Consequently, the extent of geographic and institutional disparities cannot be quantified reliably. Similar limitations in access to multidisciplinary hepatobiliary services and structured referral networks have been reported in other low- and middle-income settings [4,5,17].

Centralization of major BDI care has been consistently associated with more favorable clinical outcomes. High-volume HPB centers report lower rates of postoperative complications, biliary strictures, and reinterventions, particularly when definitive reconstruction is performed by experienced hepatobiliary surgeons rather than after previous repair attempts at nonspecialized institutions [4,5]. Mexican referral centers have reported favorable long-term outcomes following Roux-en-Y hepaticojejunostomy and complex biliary reconstruction [3,10]. These results demonstrate the capacity of selected national centers to manage complex injuries successfully but should not be interpreted as representative of treatment capacity throughout the Mexican healthcare system.

Comparative studies further suggest that delayed referral and unsuccessful repair before transfer are more frequent in resource-constrained settings and are associated with greater reconstructive complexity and lower long-term biliary patency [4,5]. Mexican series have described referral delays extending from several weeks to months after the index injury, frequently after multiple endoscopic, radiological, or surgical interventions performed at referring institutions [3,18]. Such delays may permit persistent biliary contamination, inflammation, cholangitis, and stricture formation, thereby complicating subsequent reconstruction. Nevertheless, because these observations derive mainly from tertiary referral cohorts, they may overrepresent patients with the most complex injuries and longest treatment pathways.

Variation has also been reported within the Mexican public healthcare system. Differences between institutions in the use of open and minimally invasive cholecystectomy, referral timing, previous repair attempts, and access to specialist care suggest that organizational structure and institutional experience may influence treatment pathways independently of the operative technique itself [9,10]. However, direct comparisons between public and private institutions remain limited, preventing firm conclusions regarding sector-specific differences in access or outcomes.

Taken together, the available evidence suggests that improving BDI outcomes in Mexico may require both expansion of specialized capacity and better organization of existing resources. Standardized referral criteria, early communication with HPB centers, multidisciplinary evaluation, and avoidance of nonspecialist definitive repair may reduce delays and unnecessary reinterventions. The development of prospective national or multicenter registries would also improve epidemiological surveillance, facilitate evaluation of referral pathways and outcomes, and provide a more accurate assessment of regional and institutional disparities.

Discussion

This review indicates that post-cholecystectomy BDI should not be understood solely as the consequence of an isolated technical error. Rather, it results from the interaction of anatomical complexity, inflammatory distortion, visual misperception, intraoperative judgment, surgeon experience, access to adjunctive technologies, and healthcare-system organization [6]. The principal findings are that the true epidemiological burden of BDI in Mexico remains uncertain; prevention depends primarily on reliable anatomical identification and timely modification of unsafe dissection; outcomes are closely associated with early recognition, adequate source control, and specialist reconstruction; and unequal access to diagnostic, interventional, and HPB resources appears to influence the entire care pathway [1,4,6,9].

Prevention remains the most effective strategy for reducing the clinical and socioeconomic consequences of BDI. Although advances in endoscopy, interventional radiology, and biliary reconstruction have improved treatment outcomes, even successfully managed injuries may require repeated interventions, prolonged surveillance, and multidisciplinary care [2,15]. The CVS remains the principal standardized method for confirming biliary anatomy before ductal division [2]. Importantly, failure to achieve CVS should function as a decision point rather than an invitation to continue increasingly hazardous dissection. In difficult operative conditions, subtotal cholecystectomy, fundus-first dissection, temporary drainage, conversion, or referral should be regarded as safety-oriented strategies rather than technical failure [2,6].

Adjunctive technologies may improve anatomical assessment but cannot substitute for sound surgical judgment. Intraoperative cholangiography may clarify uncertain anatomy and facilitate recognition of injury, while indocyanine green fluorescence may improve visualization of the extrahepatic biliary tree [1,12]. Their value depends on correct acquisition, interpretation, and integration into a structured intraoperative strategy. The central preventive principle is therefore not the routine use of any single technology, but avoidance of ductal division when anatomy remains uncertain.

The clinical course of BDI is strongly influenced by the timing of recognition. Intraoperative diagnosis offers the best opportunity to characterize the injury, control contamination, assess vascular involvement, and obtain specialist input. Delayed recognition may permit progression to biloma, biliary peritonitis, cholangitis, sepsis, and secondary stricture formation, frequently resulting in multiple interventions before definitive treatment [1]. The low proportion of injuries recognized intraoperatively in Mexican referral experience underscores the need to strengthen both intraoperative safety practices and postoperative vigilance [2].

Management should be individualized according to physiological stability, ductal continuity, injury level, biliary contamination, and associated vascular damage. Computed tomography is particularly useful for identifying collections and vascular or perfusion abnormalities, whereas magnetic resonance cholangiopancreatography better defines proximal biliary anatomy and the level of obstruction [1]. Endoscopic retrograde cholangiopancreatography is most effective in leaks and partial injuries with preserved ductal continuity but has a limited role in complete transection or disconnected proximal ducts [1]. These distinctions support a staged approach in which source control, biliary decompression when required, and physiological stabilization precede definitive reconstruction [1,17].

The optimal timing of reconstruction remains debated and cannot be considered independently of local inflammation, sepsis, vascular involvement, anatomical definition, and surgeon expertise [4,17]. Early repair may be appropriate when the injury is promptly recognized, contamination is controlled, and an experienced HPB surgeon is available. Conversely, delayed reconstruction may be preferable in the presence of severe inflammation, biliary sepsis, or uncertain anatomy. Available evidence suggests that the experience of the reconstructing team may be at least as important as the chronological interval to repair. Previous unsuccessful repair attempts can reduce healthy ductal tissue, increase reconstructive complexity, and compromise long-term biliary patency [4,5,15]. When appropriate expertise is unavailable, controlled drainage and prompt referral are therefore preferable to an unplanned definitive repair.

Roux-en-Y hepaticojejunostomy remains the principal reconstructive procedure for major injuries with loss of biliary continuity. Favorable long-term outcomes have been reported when reconstruction is performed using healthy, well-vascularized proximal ducts at experienced centers [3,5,10,17]. Nevertheless, late anastomotic stricture, recurrent cholangitis, secondary biliary cirrhosis, and complex re-repair remain important concerns, particularly after delayed referral or previous failed reconstruction [4,5,17]. Long-term surveillance is therefore warranted even after apparently successful treatment.

A central finding of this review is the contrast between the high-level capacity demonstrated by selected Mexican referral centers and the limited evidence regarding national access to such care. Mexican institutions have reported favorable outcomes after complex open, minimally invasive, and robotic reconstruction [3,10]. However, these results reflect the experience of specialized centers and should not be interpreted as representative of the entire healthcare system. The available national literature instead suggests delayed recognition, prolonged referral intervals, previous unsuccessful interventions, and marked institutional variation [4,9].

The principal healthcare challenge in Mexico may therefore be less the absence of effective diagnostic and reconstructive options than their unequal distribution and delayed accessibility. Patients treated initially in institutions without therapeutic endoscopy, interventional radiology, or HPB surgery may undergo repeated temporizing procedures before reaching definitive care, potentially increasing reconstructive complexity and compromising long-term outcomes [4,15]. Standardized referral criteria, early communication with regional HPB units, avoidance of nonspecialist repair, and formalized transfer pathways may provide greater population-level benefit than isolated investment in advanced technology.

Emerging technologies should also be interpreted according to their clinical context. Population-based studies have reported higher rates of BDI requiring operative intervention after robotic-assisted cholecystectomy than after conventional laparoscopy [7,8]. In contrast, robotic hepaticojejunostomy has been reported as feasible in selected patients treated by experienced reconstructive teams [3,10]. These findings should not be conflated: concerns regarding robotic cholecystectomy do not establish the ineffectiveness of robotic reconstruction, and favorable reconstructive outcomes do not demonstrate the superiority or safety of the robotic platform during routine cholecystectomy. Competency-based training, case selection, supervision, and outcome monitoring remain essential.

The principal evidence gap is the absence of a national or multicenter Mexican registry. Institutional referral cohorts cannot establish national incidence because they lack a denominator representing the total number of cholecystectomies and may overrepresent complex injuries, delayed presentations, and patients requiring reconstruction. Future studies should standardize the reporting of injury severity, recognition and referral intervals, previous interventions, reconstruction technique, biliary patency, stricture, cholangitis, reintervention, mortality, healthcare utilization, and patient-reported quality of life. Long-term outcomes are particularly important because technical patency alone may not capture persistent symptoms, repeated healthcare use, psychosocial effects, and reduced quality of life [15,18].

This review is limited by the predominance of retrospective observational studies and institutional series, substantial heterogeneity in injury classification, management, follow-up, and outcome definitions, and the concentration of Mexican evidence in tertiary referral centers [4,11,12]. The search excluded grey literature, was restricted to English, Spanish, and Portuguese publications, and did not include a formal risk-of-bias assessment. The findings should therefore be interpreted as a critical narrative synthesis rather than a quantitative comparison of treatment effectiveness.

Overall, improving BDI outcomes requires alignment between surgical safety, early recognition, multidisciplinary management, specialist reconstruction, and healthcare-system organization. In Mexico, strengthening regional referral pathways, concentrating complex reconstruction in experienced centers, standardizing outcome reporting, and establishing prospective multicenter surveillance may be more consequential than the isolated adoption of new technologies. The optimal result depends not only on selecting the appropriate intervention, but also on delivering it at the appropriate stage of injury and within the appropriate level of expertise.

## Conclusions

Post-cholecystectomy bile duct injury remains a potentially life-altering complication whose outcomes depend on more than the technical success of repair. Prevention through reliable anatomical identification, timely recognition, effective source control, and early involvement of experienced hepatopancreatobiliary teams are central to reducing morbidity and preserving long-term biliary function. In Mexico, the principal challenges appear to be the absence of national surveillance, delayed referral, and unequal access to specialized diagnostic, interventional, and reconstructive resources. Strengthening regional referral pathways, standardizing care processes, concentrating complex reconstruction in experienced centers, and developing prospective multicenter registries may therefore offer the greatest opportunity to improve outcomes at the population level.
